# This or not that: select and reject control of relational responding in rats using a blank comparison procedure with odor stimuli

**DOI:** 10.1007/s10071-024-01881-7

**Published:** 2024-06-17

**Authors:** Bobbie Faith Wolff, Mark Galizio, Katherine Bruce

**Affiliations:** Department of Psychology, UNC Wilmington, 601 S. College Rd., Wilmington, NC 28403 USA

**Keywords:** Blank comparison, Select control, Reject control, Stimulus control topography, Exclusion learning, Rats

## Abstract

The blank comparison (BLC) task was developed to assess stimulus relations in discrimination learning; that is, are subjects learning to “select” the correct stimulus (S+) or “reject” the incorrect stimulus (S-) or both? This task has been used to study exclusion learning, mostly in humans and monkeys, and the present study extends the procedure to rats. The BLC task uses an ambiguous stimulus (BLC+/-) that replaces S+ (in the presence of S-) and replaces S- (in the presence of S+). In the current experiment, four rats were trained to remove session-novel scented lids from sand-filled cups in a two-choice, simultaneous presentation procedure called the Odor Span Task (OST) before being trained on the BLC procedure using odors as the discriminative stimuli. The BLC training procedure utilized simple discrimination training (S+ and S-) and added select (S+ and BLC-) and reject (BLC+ and S-) trial types. All rats demonstrated accurate performance in sessions with both select and reject type trials. Next, BLC probe trials were interspersed in standard OST sessions to assess the form of stimulus control in the OST. Rats performed accurately on select type probe trials (similar to baseline OST performance) and also showed above chance accuracy on reject type trials. Thus, we demonstrated that rats could acquire an odor-based version of the BLC task and that both select and exclusion-based (reject) relations were active in the OST. The finding of exclusion in rats under the rigorous BLC task conditions confirms that exclusion-based responding is not limited to humans and non-human primates.

## Introduction

Discrimination learning is shown when animals consistently respond differentially to stimuli in line with the programmed contingencies. However, such successful discrimination may be based on different forms of learned relations. For example, consider a conditional discrimination procedure such as matching-to-sample with red and green stimuli. After such a discrimination has been mastered, the researcher might assume that the subject has learned to select the comparison stimulus that matches the sample (i.e., select red comparison given a red sample). But alternatively, the subject may have learned to reject the green comparison given a red sample. *Select control* is said to occur when selection of a stimulus is controlled by the relation between the sample and the correct comparison. Conversely, rejection-based, or *reject control*, occurs when selection of a stimulus is controlled by the relation between the sample and the incorrect comparison, without involvement of the correct comparison until the response occurs (McIlvane et al. 1987). These different forms of stimulus control have been termed stimulus control topographies (SCT; McIlvane 2013; McIlvane and Dube 1992; Ray 1969).

Both select and reject patterns can develop during simple and conditional discrimination training and, because multiple SCTs can produce successful discrimination, it is often difficult to determine which has been learned. When the SCT that develops is different than the researcher intended, problems of interpretation can arise. For example, in conditional discrimination training designed to produce equivalence classes, the expected classes will not emerge if reject control develops (Johnson and Sidman 1993). On the other hand, reject control can be valuable as it is the basis of learning through exclusion which is important in language and other complex skill acquisition (Wilkinson et al. 1996, 1998, 2009).

Analysis of the SCT following discrimination training requires special procedures. One methodology specifically designed to directly test what has been learned in the context of conditional discriminations is the blank comparison procedure first implemented by McIlvane and colleagues (1987). This procedure uses an ambiguous (i.e., blank) stimulus, the reinforcement status of which depends upon the stimulus it is paired with, to directly assess the presence of select- and reject-controlling relations. If the blank comparison is presented along with a negative comparison (S-), selection of the blank is reinforced, but if the blank is presented along with a positive comparison (S+), selection of the S+ and not the blank is reinforced. Correct responses on trials that include the blank comparison and the trained S+ show that select control relations are present. Correct responses on trials that include the blank comparison and the trained S- show that reject stimulus control relations are present. Conversely, failure to make correct responses on trials including either the S+ or S- stimulus indicates that select- or reject-controlling relations are not present, respectively. This procedure has been used effectively to isolate SCTs in conditional discriminations using human participants (e.g., Costa et al. 2001; McIlvane et al. 1987; Wilkinson & McIlvane, 1997).

There has been interest in developing variations of the blank comparison procedure in nonhumans—particularly to address the question of whether nonhumans are capable of learning through exclusion (the selection of a correct alternative by elimination of the other incorrect alternatives). While there are a number of demonstrations of exclusion across a broad range of nonhuman species including chimpanzees (Beran and Washburn 2002), capuchin monkeys (Goulart et al. 2005; Jiménez et al. 2017), California sealions (Kastak & Schusterman, 2002; Biolsi and Woo 2022 for a review), dogs (Aust et al. 2008; Kaminski et al. 2004; Pilley & Reid, 2011; Zaine et al. 2016), rats (de Souza and Schmidt 2014), pigeons (Clement and Zentall 2003), keas (O’Hara et al. 2016) and bees (Scienza et al. 2019), most of these studies did not use the blank comparison procedure. Rather, they trained subjects on simple or conditional discriminations and then demonstrated exclusion by selection of a novel stimulus when paired with an S- on a trial. These studies are generally taken as supporting the claim that exclusion is widespread across species; however, interpretation is complicated by the commonly observed tendency to approach novel stimuli.

Two of the studies cited above did use a blank comparison procedure (Goulart et al. 2005; Scienza et al. 2019). Goulart et al. trained two capuchin monkeys on a simultaneous simple discrimination with two visual stimuli (i.e., a triangle S+ and a horizontal line S-) with a blank comparison (white square) intermixed on some trials. On blank comparison trials, when the blank was paired with S-, selection of the blank was reinforced, but when paired with S+, selection of the blank was not reinforced. Once this discrimination was acquired, probe trials with novel stimuli paired with the triangle and horizontal line were introduced. Both monkeys showed high accuracy on the probe trials suggesting that both select and reject SCTs had been acquired through the blank comparison training. Scienza et al. (2019) used similar procedures with stingless bees and showed both select and reject responding, but there are no other published studies using the blank comparison methodology in nonhumans.

Both the Goulart et al. (2005) and Scienza et al. (2019) studies used simple discrimination procedures. But given that the blank comparison methodology has yielded clear evidence of select and reject controlling relations in humans in conditional discrimination procedures, it would be of interest to determine whether this also applies to nonhumans. Thus, one purpose of the present study was to develop a variation of the blank comparison procedure that could be used to study conditional discrimination and a second was to extend the procedure to a new species, rats. Rodents are commonly used in behavioral and neuroscience research, so it is somewhat surprising that the blank comparison procedure has not been used in rats. There are several studies that suggest that reject control may develop in rats. De Souza and Schmidt (2014) trained rats on a simple discrimination with a triangle (S+) and a vertical line (S-). They then administered exclusion probe trials in which the vertical line S- was presented along with a novel visual stimulus and five of six rats selected the novel stimulus, suggesting exclusion. As a control for rats’ tendency to respond to novel stimuli, they included probe trials that paired a novel stimulus with the triangle S+ and found nearly exclusive responding to S+. This finding suggests that responding to the novel stimulus on exclusion probes was controlled by a reject relation rather than novelty selection, but the finding could also be explained by strong S+ control overriding novelty preference.

Additional analysis of SCTs in rats comes from research using the Odor Span Task (OST), an incremental non-matching-to-samples procedure (Dudchenko et al. 2000). Food is obtained by digging in scented sand or removing scented lids from cups placed in a large arena. Selection of an odor that is novel to the session is reinforced (S+) but previously encountered comparison odors (S-) are not. On each trial, one session-novel odor S+ is introduced along with one or more previously presented S- odors. The session-novel odor presented on each trial serves both as the reinforced comparison stimulus and as a sample to be remembered on future trials, hence the procedure term “incrementing non-matching to samples.” Rats show rapid acquisition with this procedure and are capable of accurate responding even with 70 or more odors to remember within a session (April et al. 2013). These remarkable features raise the question of whether accurate responding on the OST is based on select control (remembering that a specific comparison odor was presented longer ago than the current session and selecting it) or reject control (remembering that a specific comparison odor was presented recently and rejecting it) or some combination of SCTs.

April et al. (2013) examined this question by analyzing the pattern of visits made during OST trials where a visit was defined as a close approach to a scented lid without selecting it. Did rats attain high accuracy by immediately recognizing the session-novel S+ (select control) or responding based on relative familiarity with each of the available comparisons by first rejecting those already recently encountered (reject control)? If relative familiarity were controlling responding, it was hypothesized that the animals would generally visit all or most of the available stimuli before making a response and that this would mean at least occasionally visiting the S+ stimulus as a part of that process. However, the data showed that the animals rarely visited every available S- stimulus before responding to the S+. Similarly, also using the OST, Galizio et al. (2016) found that rats frequently visited S- comparison stimuli but made virtually no visits to the S+ stimuli. That is, when a session-novel S+ was encountered, rats responded immediately, thus ending the trial, whereas when a previously presented S- odor was encountered, rats generally sampled it on a visit and moved on to another stimulus cup. These data suggest selection-based control.

It remains to be determined whether reject control also develops in the OST; special techniques, such as the blank comparison procedure, are needed to isolate select and reject control. Thus, the present experiment had three main purposes: first, as noted above, to develop a variation of the blank comparison procedure suitable for rats; second, to extend the blank comparison procedure to a conditional discrimination; and third, to use this procedure to examine sources of control in an incrementing non-matching-to-samples task, the Odor Span Task.

## Method

### Subjects

Four male albino Sprague-Dawley rats were maintained at 85–90% of their free feeding weight with free access to water in their home cages. Rats were individually housed in a vivarium on a reversed 12-hour light-dark cycle and tested during the dark cycle. Prior to starting the study, two rats (B13 and B15) had been trained to lever press, one animal (B14) to nose poke in an operant chamber, and both A13 and B15 had OST training prior to the present experiment. Rats were between 5 and 15 months of age when beginning this study. Animal care and procedures were approved by the UNC Wilmington Animal Care and Use Committee.

### Apparatus

**Fig. 1:**
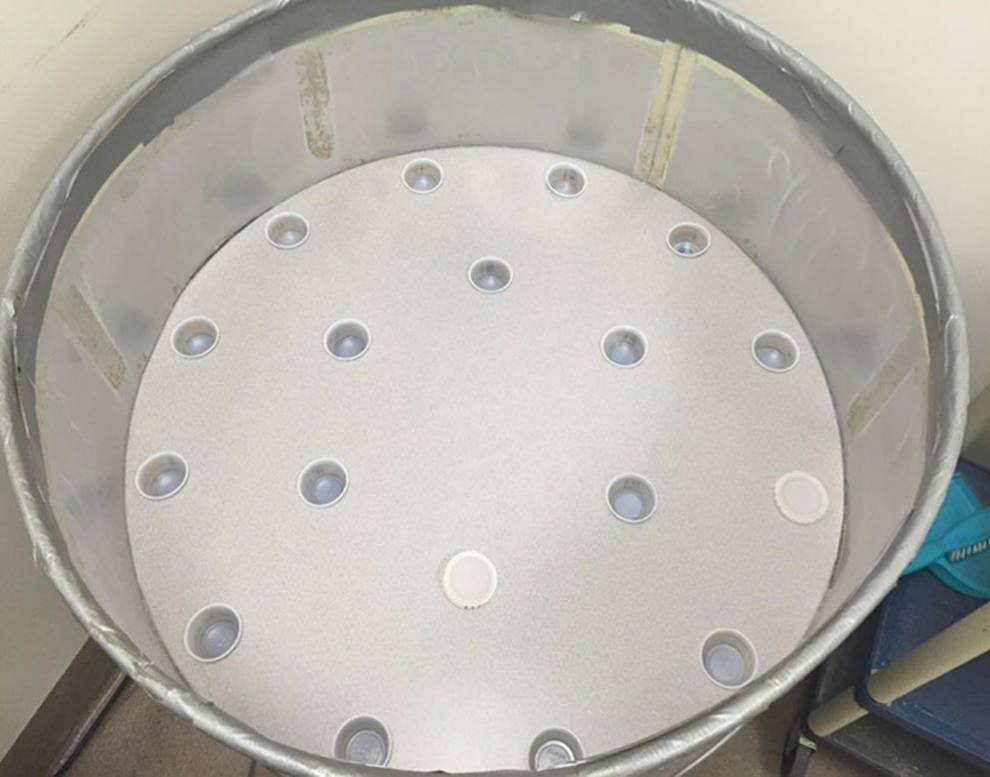
Arena testing apparatus. *Note* In the testing arena, there are two circular arrays (rings) in which the stimuli can be placed. In the outer ring, there are 12 equidistant holes around the perimeter. Note that a cup with lid is in the 4 o’clock position. There are six equidistant holes in the center ring; there is a cup with lid in the 6 o’clock position

Training and testing of all subjects took place in an open field circular arena (94 cm diameter), surrounded by metal baffling (32 cm high). The arena floor contained 18 holes (5 cm in diameter, 13.3 cm apart) arranged in two circular arrays as displayed in Fig. 1. Plastic stimulus cups (60 ml) were placed in each of the 18 holes during sessions. A rectangular plastic holding cage was used to house subjects between trials. White noise (ca. 74 dB) was presented throughout the sessions and trials were recorded using a digital video camera. In order to avoid cuing, the experimenter stood out of view of the rat during trials and observed the rat’s behavior on the video monitor.

### Stimuli

Olfactory stimuli were common spices and teas presented by covering sand filled stimulus cups with opaque, scented plastic lids. The lids were stored in plastic containers filled with their respective odorants for a minimum of two weeks prior to being used and all odorants were replaced every six weeks to maintain their potency. Odorants were purchased from the Great American Spice Co. and Davidson’s Tea Company. Odorants used in the Odor Span Task included allspice, anise, bay, caraway, celery, cinnamon, clove, coriander, cumin, dill, fennel, fenugreek, garlic, ginger, marjoram, mustard, nutmeg, onion, oregano, rosemary, sage, savory, spinach, sumac, and thyme. Basil, carob, chamomile tea, English Breakfast tea, lavender, parsley, and sassafras odorants were only used for simple discrimination and blank comparison trials.

### Procedure

#### Shaping, habituation, and pre-training

Subjects were initially habituated to the arena by placing them in the apparatus with all 18 holes filled with plastic cups that were each half-filled with sand. Each cup was baited with one sucrose pellet (45 mg Bio Serv) placed in the sand. Habituation sessions ended when the subject was reliably eating all pellets. Lid-removal shaping followed; in these sessions, the arena contained 17 empty cups and 1 baited cup. An unscented plastic lid was placed so that it partially covered the baited cup. As subjects successfully moved the lid and retrieved the pellet, the lid was gradually re-positioned to cover more of the cup until it eventually covered the entire cup. The baited cup was then moved to a new random location each trial. Once subjects were reliably removing lids and retrieving pellets (at least 24/25 lids removed from fully covered cups within a session), Odor Span Task (OST) training began. Shaping and pre-training procedures typically required about five sessions. Subjects were tested five days per week (typically Monday through Friday) throughout the study.

#### Odor span task training

In initial training, a “resetting” OST was conducted. On Trial 1, a baited stimulus cup was placed in one of the 18 arena holes and covered with a scented lid. All of the other holes were filled with empty plastic cups. The subject was then placed in the center of the arena (rats were placed facing the same direction on all trials) and allowed to move freely about the arena until removing the lid from the baited cup and obtaining reinforcement. The experimenter then moved the subject to the holding cage for the approximately 30-s inter-trial interval. On Trial 2, a new baited cup with a session-novel odorized lid (S+) was placed in the arena along with an unbaited cup scented with the previously presented odor (S-). To avoid the rat’s own scent serving as a possible cue, lids were used only once per session. Removal of the S+ lid was scored as a correct response; removal of the S- lid was scored as an error and the trial continued until the S+ was selected. This process continued with one session-novel S+ comparison presented on each trial and the number of S- comparison stimuli increasing by one in each trial until an error was made. After an error, the session-novel S+ was presented alone on the next trial (the session “reset”) and the incrementing procedure continued until the session terminated after 25 trials, thus exposing the rat to 25 different odors. The position of cups in the arena was determined randomly as was the order of selection of S+ odors each session. Training continued until at least one session with a longest run of 10 consecutive correct trials or until two consecutive sessions with a longest run of five or more occurred. This phase typically required 5–10 sessions.

When this criterion was met, OST training proper began; the procedure was changed to hold the number of comparison stimuli present in the arena to two (see Mathews et al., 2018). After Trial 1, each trial included one baited session-novel (S+) scent and one S- odor selected randomly from the set of odors previously presented during that session. Rats were tested on this procedure until their average accuracy on the OST for the most recent five testing days was at least 72%. When this criterion was met, a simple discrimination (SD) was trained to provide a basis for the BLC procedure that followed.

#### Simple discrimination (SD) training

All subjects were trained on a two-choice simple discrimination procedure with one pair of odor stimuli that were not used in the OST procedure (see Table 1). Selection of one scent (SD+) was always reinforced and the other (SD-) was never baited throughout the experiment. Simple discrimination sessions (25 trials each) alternated with OST sessions across days; session type (OST or SD) was randomly chosen with the constraint that no session type was conducted for more than two consecutive days. Rats were tested for a minimum of 10 sessions in this alternation procedure, with at least five sessions of each type. The criteria to advance to mixed trials were an average OST accuracy of at least 72% correct for five sessions (and no session less than 64%) and at least 80% correct on the SD sessions over the last five days.

**Table 1:** Simple discrimination and blank comparison odors assigned to each rat

Rat	SD+	SD-	BLC+/-
B13	Sassafras	Carob	Basil
B14	Lemongrass	Carob	Lavender
B15	Chamomile Tea	Parsley	Rooibos Tea
A13	Sassafras	Carob	English Breakfast Tea

Rats then moved to mixed trials training; six simple discrimination trials were added at the end of the 25 OST trials each day. After a five-session minimum, a criterion of a five-session mean of 72% or greater correct on OST trials with no session less than 64%, and a five-session simple discrimination average of 80% or greater, blank comparison (BLC) training began.

#### BLC training

Subjects were trained on the blank comparison (BLC+/-) procedure in several steps, all of which included the introduction of a BLC odor, the reinforcement status of which always depended upon the odor with which it was paired. Each rat was assigned a BLC+/- scent not previously used in the OST (see Table 1 for each animal’s assigned scents). This allowed for the construction of select (SD+/BLC-) and reject (BLC+/SD-) training trials. These were conducted like SD trials except that on BLC trials, the two stimulus cups were never more than one cup location apart to facilitate learning of the conditional discrimination and to better approximate the simultaneous presentation achieved with visual stimuli by Goulart et al. (2005).

BLC training sessions were 30 trials. When we began training the first rat in the protocol (B13), the first 20 trials were BLC (reject and select trials evenly intermixed) with 10 SD trials at the end. After 10 sessions of inaccurate responding and B13 making little contact with the BLC odor, we changed to block training that included overexposure to select trials to increase his inspection (visits) of the BLC odor. Thus, in this training, the first 20 trials were select (SD+/BLC-) and the last 10 trials were reject (BLC+/SD-) trials. Criteria to complete this phase included a five-session minimum and 70% correct average for both select and reject trials for the last five sessions. We continued this training protocol for the other three rats when they began the experiment. All of the animals met the performance criteria by the end of the first five days of block training.

After this initial BLC training in blocks, BLC training continued with trial types interspersed. As before, the first 20 trials paired the SD+ used in simple discrimination training with the BLC stimulus serving as the S-. However, now the last 10 trials were five select (SD+/BLC-) and five reject (BLC+/SD-) type trials presented in a random order. After five sessions, the initial block of select trials was reduced from 20 to 15 and the final 15 trials included eight select trials and seven reject trials presented in a random order. As noted above, this procedure was used to increase subjects’ exposure to the select trial contingency. Acquisition of the BLC task was demonstrated when the rats were reliably responding to the BLC in the presence of the SD- on reject trials and responding to the SD+ in the presence of the BLC on select trials. Further, rats were required to maintain this discrimination when select trials were randomly interspersed with reject trials in the final testing block of each session. Accurate responding on these interspersed select trials would show that the animals had learned the stimulus relations and were not responding on the basis of trial order.

The interspersed BLC training sessions alternated with OST/SD sessions throughout the five testing days each week to ensure that OST performances remained accurate. Sessions were conducted such that four days of the week were used for BLC training sessions and one day a week was used for OST/SD. This phase was complete when rats met the following criteria: (1) 80% correct or higher accuracy for both select and reject trial types during the final block of the session over the last five BLC sessions while (2) maintaining above 80% accuracy on both OST and SD trials during that same period (2–4 OST/SD sessions depending on the subject).

#### Blank comparison probe trials in the OST (BLC in OST)

In this phase, probe trials using the BLC were introduced to directly assess select and reject control in the OST. These sessions began with six regular BLC trials (three select and three reject presented in a random order) in order to verify that relations between the SD+, SD-, and BLC+/- odors were intact. If the rat responded correctly on at least 5/6 of these trials (83% correct), then a BLC in OST session was conducted. If this criterion was not met, OST trials were not programmed for that session but instead a BLC training session was conducted.

The BLC in OST sessions were 25 trials, consisting of 17 OST trials plus eight BLC probe trials interspersed. These included six reject (BLC/S-) trials on which the S- was a previously presented OST odor paired with the BLC odor and two select (S+/BLC) trials on which the S+ was a session-novel odor paired with the BLC stimulus. For example, if clove had been presented previously on an OST trial, then a BLC/S- probe trial (reject) could present clove and the BLC in the arena with selection of the BLC reinforced. A BLC/S+ trial paired a session-novel stimulus, say thyme, with the BLC with selection of thyme reinforced (select). Because select responding had already been demonstrated in OST performance (see April et al. 2013; Galizio et al. 2016), we oversampled reject probe trials to see if rats would perform accurately on this type of trial.

The BLC trials were quasi-randomly interspersed with some restrictions. First, the first trial that included the BLC was always a reject (BLC+/S-) trial. Second, no session included more than two consecutive BLC+/- trials of either type. Twenty-five sessions under these conditions were conducted for each rat. Evidence of reject and select control on BLC trials was measured via stimulus visits and final response (lid removal) in BLC trials. Visits were operationalized as the animal’s nose coming within 1 cm of a scented lid, but without lid removal.

### Control procedures

#### Retention interval control

To keep the retention interval relatively consistent on reject trials in the BLC in OST procedure, the S- stimulus odor was randomly selected from the previous five OST trials.

#### Odor detection control

To control for the possibility that animals might respond to a stimulus by detecting a sucrose pellet underneath the scented lids, some trials were conducted with the S+ stimulus cup unbaited. Correct responses were reinforced manually by the experimenter with a pellet delivered to the cup through a long straw inserted in the arena. This control was used on all BLC trials during BLC in OST sessions and in eight randomly selected BLC trials of every session during BLC training. In addition, to ensure that rats were not responding on the basis of the scent of previously encountered sand cups rather than on the basis of lid scent, session-fresh cups were used on some trials. All BLC trials were run with this control in place.

#### Inter-rater reliability

Data were collected manually. Inter-rater reliability was assessed by rescoring a subset of the sessions that were video recorded (*n* = 39). Percent agreement was high with 99% agreement for lid removal and 90% for visits.

## Results

### OST and SD training

Table 2 shows the numbers of Odor Span Task (OST) and simple discrimination (SD) training sessions required to meet criteria to advance to BLC Training for each subject. Subjects met mastery criteria for the OST in 37 or fewer training session and for SD training in 11–22 sessions prior to beginning Blank Comparison Task (BLC) training.

**Table 2:** Number of sessions completed by each subject

Rat	OST and SD Training			BLC Training		BLC in OST
	OST Alone Training Sessions	SD Alone Training Sessions	Mixed OST and SD Sessions	BLC Training Sessions	OST Sessions	OST with BLC Interspersed
B13	37	5	17	18	7	25
B14	21	6	5	28	7	25
B15	3	6	6	24	6	25
A13	7	6	5	41	10	25

*Note* B15 and A13 had already learned the OST in another experiment, thus requiring only a few OST alone training sessions to meet criteria to move to SD training. Mixed Trials included 25 OST trials with 6 SD trials at end

### Blank comparison training

**Fig. 2:**
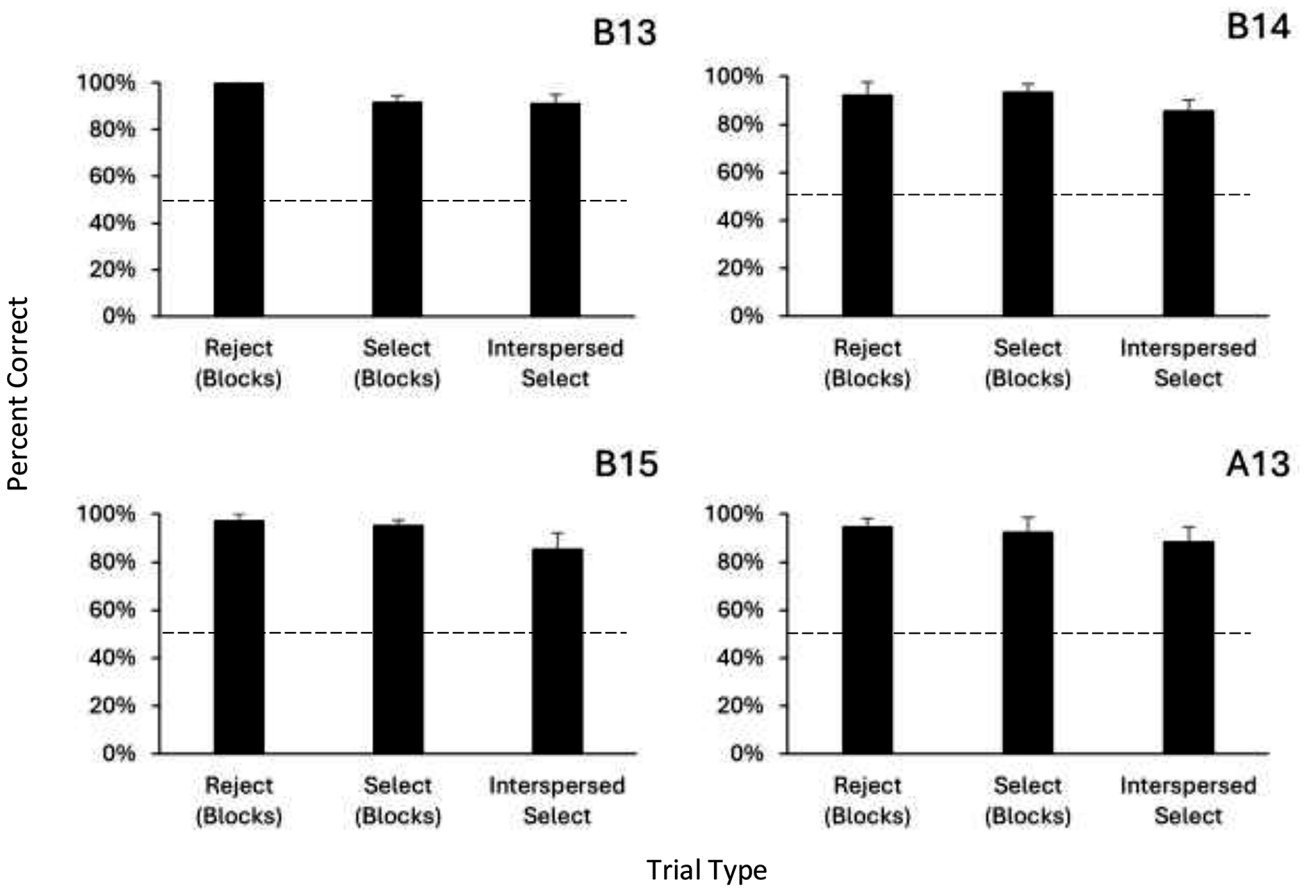
Percent correct responses by each subject in blocked reject, select, and interspersed select trials in the last five sessions of BLC training. *Note* Error bars are standard error of the mean. Dashed line indicates chance responding. All means significantly above chance (*p* < .001, binomial test)

Percent correct responses on select (SD+/BLC-) and reject (BLC+/SD-) trials during initial blocks of the session, as well as on the select trials randomly interspersed among the reject trials in the final session block, were used to determine acquisition of BLC training. As seen in Table 2, rats required 18–41 sessions of BLC training to acquire the task. Percent correct responses for the last five sessions of BLC training are presented for each rat in Fig. 2. All four rats maintained highly accurate performances on reject, select, and interspersed select trials, showing acquisition of the BLC Task; all rats showed individual performances above 85% correct and all performances were significantly above chance (all binomials, *p* < .001).

**Fig. 3:**
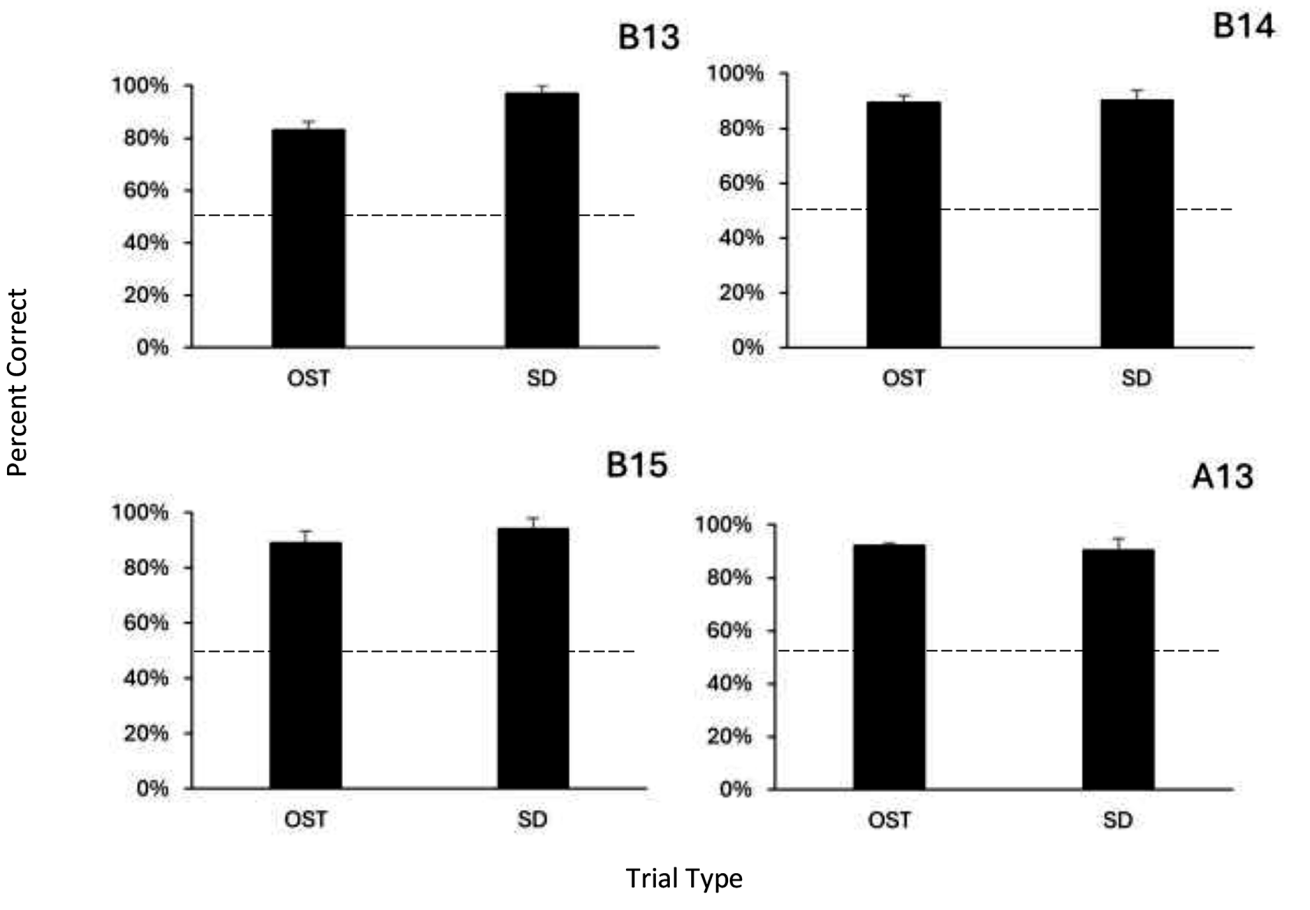
Percent correct responding by each subject on OST and SD sessions during BLC training. *Note* Error bars are standard error of the mean. Dashed line indicates chance responding. All means significantly above chance (*p* < .001, binomial test)

While the rats were learning the BLC procedure, they maintained high accuracy on the OST and SD tasks; rats completed 6–10 days of OST/SD sessions while BLC training was going on (see Table 2). Figure 3 presents percent correct responding on the OST and SD tasks; all four rats showed high accuracy on both tasks (OST: *M* = 88.7%, *SE* = 1.9; SD: *M =* 93.2%, *SE* = 1.6), all significantly above chance performance (all binomials, *p* < .001. Together, the data presented in Figs. 2 and 3 show that all animals were able to acquire and perform the BLC task without disruption to their performance on the other two tasks.

### BLC in OST

**Fig. 4:**
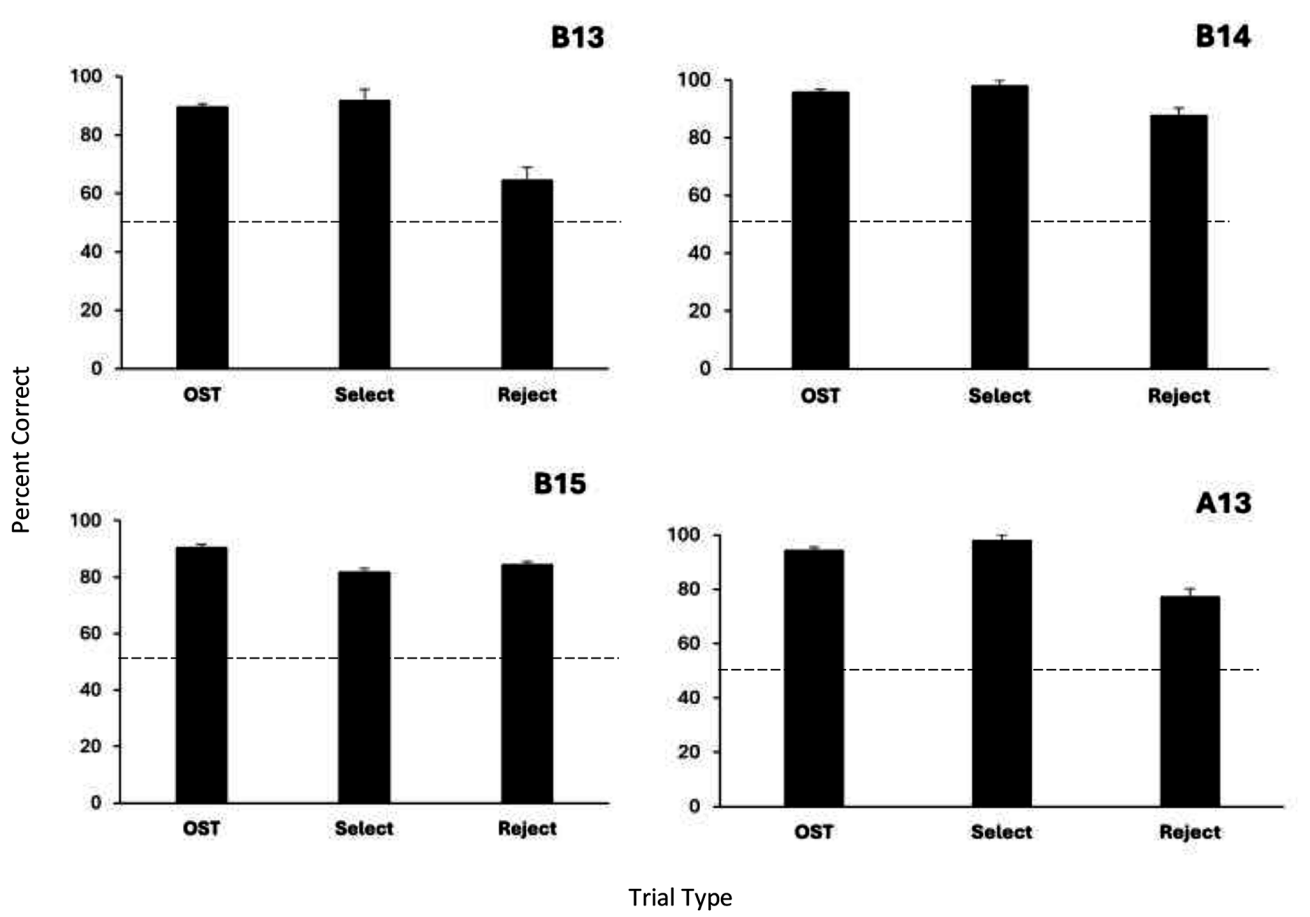
Precent correct responding by each subject on OST, select, and reject trials across all 25 BLC in OST sessions. *Note *Error bars are standard error of the mean. Dashed line indicates chance responding. All means significantly above chance (*p* < .001, binomial test)

Figure 4 shows percent correct responding across the 25 BLC in OST sessions. For each rat, there were a total of 425 typical OST trials along with 50 select and 150 reject probe trials. Overall, the data show accurate performance across rats for all trial types, with all performances above chance (all binomials, *p* < .001). Accuracy for two rats (B14 and B15) was very high (> 80% correct) and nearly equivalent across OST trials and both probe types, but Rats B13 and A13 showed somewhat lower accuracy on the reject probes. In particular, Rat B13, had just over 60% correct on the reject probes which, although above chance, was substantially lower than his OST and select probe performances. Rat A13 showed close to 80% correct on the reject probes, but his OST and select probe accuracy was considerably higher (e.g., over 90%).

We noted a unique pattern of responding on reject trials during the BLC in OST phase. Rats typically make few S+ visits in the OST; they immediately respond to the session-novel stimulus rather than sampling the odor and moving on. However, on reject trial types, we observed that the rats were very likely to sample the BLC+, move to the other comparison, then return to the BLC+. We labeled this pattern *dual visits*. Table 3 presents the percentage of trials with dual visits for each trial type- reject, select and OST. For OST trials, dual visits were only observed for one of the rats (A13) and only on less than 1% of trials. Further, no dual visits were observed for any of the rats on select trials. Dual visits were very common on reject trials for all rats, ranging from 33 to 80% of these trial types. It is likely that dual visits are a consequence of the BLC procedure; the BLC is an ambiguous stimulus, and its reinforcement likelihood depends on the comparison stimulus. This will be discussed further below.

**Table 3:** Percentage of trials with dual visits during the 25 BLC in OST sessions

Rat	Select Trials (S+ vs. BLC-)	Reject Trials (BLC+ vs. S-)	OST Trials (S+ vs. S-)
B13	0%	38.67%	0%
B14	0%	44.67%	0%
B15	0%	64%	0%
A13	0%	65.33%	0.71%

## Discussion

One major purpose of the present study was to determine whether stimulus control topography could be assessed in rats using a variation of the BLC task with olfactory stimuli. After learning a simple discrimination between two odors (SD+/SD-), rats successfully learned to respond to SD+ when the blank comparison odor (BLC-) was present and to respond to the blank comparison (BLC+) when SD- was present. Although 18–41 sessions of training were required, each rat acquired the task and demonstrated accurate responding in both select and reject contexts. Performances remained accurate when BLC trials were interspersed with SD trials, indicating that behavior was not under the control of timing or trial order. Thus, both select and reject stimulus control were demonstrated with a simple discrimination in rats using the olfactory variation of the BLC task.

Further aims were to use the BLC task to evaluate the form of stimulus control in the OST. Here the BLC stimulus was introduced on probe trials and rats performed accurately on both select and reject probe trials. On select trials, when BLC- was presented along with a session-novel S+, all four rats showed high accuracy selecting the S+. Indeed, performance was no different on these BLC- trials than on regular OST trials providing strong evidence of select control. On reject trials, when BLC+ was presented along with an S- odor, responding to the BLC+ was above chance in all four animals, but was somewhat lower than accuracy on select trials in two of the rats. Reject probe trials were overrepresented during this phase which could have biased the rats to respond to the BLC. However, the high accuracies on select probe trials (which require *not* responding to the BLC) rule out such an account. Thus, both select and reject control was demonstrated to be active in the OST.

Although accurate responding was evident on both select and reject trials in the OST, there were noteworthy differences in the specifics of the rats’ behavior. On typical OST trials, rats frequently visit the S- stimulus lid without responding, but nearly always respond immediately upon approaching the novel S+ stimulus (April et al. 2013; Galizio et al. 2016). In the present study, rats consistently responded to the session-novel stimulus upon encountering it regardless of whether S- was an odor previously presented in the OST or the blank comparison on select trials. However, the topography on reject probe trials was quite different. Here we observed a high rate of dual visits where rats sampled both the S- and the BLC+ before making a response. The contextual control required in the BLC task likely provides an explanation for this difference. On OST and select trials, rats responded to the S+ odor when they encountered it, and if the S+ lid was the first to be encountered, there would be no opportunity for an S- visit on that trial. In contrast, encountering the blank comparison first on both select and reject trials is likely to result in a visit, that is, whether it functions as BLC+ or BLC- depends on the other comparison stimulus in the array. On a reject trial (BLC+/S-), this response pattern would result in dual visits: a visit to the BLC, followed by a visit to the other cup (S-), then a return to respond to the BLC. However, if it were a select trial (S+/BLC-) and the rat encountered the BLC first, he would likely visit it, then move to the S+ and respond.

A behavioral pattern like dual visitation has so far not been specifically described in the BLC Task literature, but it is possible that this is because of the differences in requiring a touch response to closely grouped visual stimuli (Goulart et al. 2005; McIlvane et al. 1987) as opposed to the arrangement typical of rodent odor tasks which requires substantial locomotor behavior (April et al. 2013; Galizio et al. 2016). Related, subjects experiencing a visual task can sample both comparisons without obvious locomotion, while that is required for the OST. Eye-tracking technology could be used for observation of this type of behavior (Hopper et al. 2020).

In summary, an odor-based version of the BLC Task identified both select and reject controlling relations in rats. The observation of reject control extends previous findings of exclusion in rats (De Souza and Schmidt 2014) to a conditional discrimination task (the OST). It is also the first demonstration of exclusion in rats using the BLC procedure which is particularly important as it eliminates novelty preference as an alternative account.

## Data Availability

The data that support the findings of this study are available from the corresponding author (K.B.) upon reasonable request.

## References

[CR1] April LB, Bruce K, Galizio M (2013) The magic number 70 (plus or minus 20): variables determining performance in the rodent odor span task. Learn Motiv 44:143–158. 10.1016/j.lmot.2013.03.001 23729864 10.1016/j.lmot.2013.03.001 PMC3665427

[CR2] Aust U, Range F, Steurer M, Huber L (2008) Inferential reasoning by exclusion in pigeons, dogs, and humans. Anim Cogn 11(4):587–597. 10.1007/s10071-008-0149-0 18309524 10.1007/s10071-008-0149-0

[CR3] Beran M, Washburn D (2002) Chimpanzee responding during conditional matching-to-sample: control by exclusion. J Exp Anal Behav 78(3):497–508. 10.1901/jeab.2002.78-497 12507016 10.1901/jeab.2002.78-497 PMC1284912

[CR4] Biolsi KL, Woo KL (2022) Equivalence classification, learning by exclusion, and long-term memory in pinnipeds: cognitive mechanisms demonstrated through research with subjects under human care and in the field. Anim Cogn 25(5):1077–1090. 10.1007/s10071-022-01658-w 35900682 10.1007/s10071-022-01658-w

[CR5] Clement ST, Zentall RT (2003) Choice based on exclusion in pigeons. Psychon Bull Rev 10(4):959–964. 10.3758/bf03196558 15000545 10.3758/bf03196558

[CR6] Costa ARA, Wilkinson KM, McIlvane WJ, Souza DDGD (2001) Emergent word-object mapping by children: further studies using the blank comparison technique. Psychol Record 51(3):343–355. 10.1007/BF03395403

[CR7] De Souza FM, Schmidt A (2014) Responding by exclusion in Wistar rats in a simultaneous visual discrimination task. J Exp Anal Behav 102(3):346–352. 10.1002/jeab.106 25220019 10.1002/jeab.106

[CR8] Dudchenko AP, Wood RE, Eichenbaum H (2000) Neurotoxic hippocampal lesions have no effect on odor span and little effect on odor recognition memory but produce significant impairments on spatial span, recognition, and alternation. J Neurosci 20(8):2964–2977. 10.1523/JNEUROSCI.20-08-02964.2000 10751449 10.1523/JNEUROSCI.20-08-02964.2000 PMC6772220

[CR9] Galizio M, April B, Deal M, Hawkey A, Panoz-Brown D, Prichard A, Bruce K (2016) Behavioral pharmacology of the odor span task: effects of flunitrazepam, ketamine, methamphetamine, and methylphenidate. J Exp Anal Behav 106:173–194. 10.1002/jeab.224 27747877 10.1002/jeab.224 PMC5821115

[CR10] Goulart KRP, Mendonca BM, Barros SR, Galvão FO, McIlvane JW (2005) A note on select – and reject -controlling relations in the simple discrimination of capuchin monkeys (Cebus apella). Behav Processes 69(3):295–302. 10.1016/j.beproc.2004.12.005 15896528 10.1016/j.beproc.2004.12.005

[CR11] Hopper ML, Gulli AR, Howard HL, Kano F, Krupenye C, Paukner A (2020) The application of noninvasive, restraint-free eye-tracking methods for use with nonhuman primates. Behav Res Methods. 10.3758/s13428-020-01465-6 10.3758/s13428-020-01465-6 32935327

[CR12] Jiménez OLÉ, Brino FLA, Goulart KRP, Galvão FO (2017) Simple discrimination by exclusion in infant capuchin monkeys. Psychol Neurosci 10(1):91–100. 10.1037/pne0000077

[CR13] Johnson C, Sidman M (1993) Conditional discrimination and equivalence relations: control by negative stimuli. J Exp Anal Behav 59(2):333–347. 10.1901/jeab.1993.59-333 16812689 10.1901/jeab.1993.59-333 PMC1322046

[CR14] Kaminski J, Call J, Fishcher J (2004) Word learning in a domestic dog: evidence for fast mapping. Science 304(5677):1682–1683. 10.1126/science.1097859 15192233 10.1126/science.1097859

[CR15] Kastak RC, Shusterman JR (2002) Sea lions and equivalence expanding classes by exclusion. J Exp Anal Behav 78(3):449–465. 10.1901/jeab.2002.78-449 12507014 10.1901/jeab.2002.78-449 PMC1284910

[CR16] McIlvane WJ (2013) Simple and complex discrimination learning. In: Madden GJ, Dube WV, Hackenberg TD, Hanley GP, Lattal KA (eds) APA handbook of behavior analysis, Vol. 2. Translating principles into practice. American Psychological Association, pp 129–163. 10.1037/13938-006

[CR17] McIlvane JW, Dube VW (1992) On terms: stimulus control shaping and stimulus control topographies. Behav Analyst 15(1):89–94. 10.1007/BF03392591 10.1007/BF03392591 PMC2733401 22478119

[CR18] McIlvane JW, Kledaras BJ, Munson CL, King JAK, De Rose CJ, Stoddard TL (1987) Controlling relations in conditional discrimination and matching by exclusion. J Exp Anal Behav 48(2):187–208. 10.1901/jeab.1987.48-187 3681184 10.1901/jeab.1987.48-187 PMC1338725

[CR19] O’Hara M, Schwing R, Federspiel I, Gajdon GK, Huber L (2016) Reasoning by exclusion in the kea (*Nestor notabilis)*. Anim Cogn 19:965–975. 10.1007/s10071-016-0998-x 27209174 10.1007/s10071-016-0998-x PMC4967098

[CR20] Pilley WJ, Reid KA (2011) Border collie comprehends object names as verbal referents. Behav Processes 86(2):184–195. 10.1016/j.beproc.2010.11.007 10.1016/j.beproc.2010.11.007 21145379

[CR21] Ray AB (1969) Selective attention: the effects of combining stimuli which control incompatible behavior. J Exp Anal Behav 12(4):539–550. 10.1901/jeab.1969.12-539 16811373 10.1901/jeab.1969.12-539 PMC1338645

[CR22] Scienza L, Pinheiro de Carvalho M, Machado A, Moreno MA, Biscassi N, das Gracas, de Souza D (2019) Simple discrimination in stingless bees (*Melipona quadrifasciata*): probing for select- and reject- stimulus control. J Exp Anal Behav 112(1):74–87. 10.1371/journal.pone.0228161 10.1002/jeab.531 31254277

[CR26] Wilkinson KM, McIlvane WJ (1997) Blank comparison analysis of emergent symbolic mapping by young children. J Exp Child Psychol 67:115–130. 10.1006/jecp.1997.2402 9388802 10.1006/jecp.1997.2402

[CR24] Wilkinson MK, Dube VW, McIlvane JW (1996) A cross disciplinary perspective on studies of rapid word mapping in psycholinguistics and behavior analysis. Dev Rev 16:125–148. 10.1006/drev.1996.0005

[CR25] Wilkinson MK, Dube VW, McIlvane JW (1998) Fast mapping and exclusion (emergent matching) in developmental language, behavior analysis, and animal cognition research. Psychol Record 48(3):407–422. 10.1007/BF03395281

[CR27] Wilkinson KM, Rosenquist C, McIlvane WJ (2009) Exclusion learning and emergent symbolic category formation in individuals with severe language impairments and intellectual disabilities. Psychol Record 59:187–205. 10.1007/BF03395658 10.1007/bf03395658 PMC2818096 20148195

[CR28] Zaine I, Domeniconi C, De Rose CJ (2016) Exclusion performance and learning by exclusion in dogs. J Exp Anal Behav 105(3):362–374. 10.1002/jeab.209 27193241 10.1002/jeab.209

